# Comment on Manole et al. Primary Pericardial Synovial Sarcoma: A Case Report and Literature Review. *Diagnostics* 2022, *12*, 158

**DOI:** 10.3390/diagnostics14101012

**Published:** 2024-05-15

**Authors:** Tomonori Kawasaki, Jiro Ichikawa, Hiroki Imada, Satoshi Kanno, Kojiro Onohara

**Affiliations:** 1 Department of Pathology, Saitama Medical University International Medical Center, Hidaka 350-1298, Saitama, Japan; tomo.kawasaki.14@gmail.com (T.K.); kanno820@saitama-med.ac.jp (S.K.); 2 Department of Orthopaedic Surgery, Interdisciplinary Graduate School of Medicine, University of Yamanashi, Chuo 409-3898, Yamanashi, Japan; 3 Department of Pathology, Saitama Medical Center, Saitama Medical University, Kawagoe 350-8585, Saitama, Japan; hirokickman@gmail.com; 4 Department of Radiology, Interdisciplinary Graduate School of Medicine, University of Yamanashi, Chuo 409-3898, Yamanashi, Japan; konohara@yamanashi.ac.jp

**Keywords:** diagnosis, fluorescence in situ hybridization, immunohistochemistry, SS18::SSX, SSX, synovial sarcoma

## 1. Introduction

With great interest, we read the article by Manole et al. titled “Primary Pericardial Synovial Sarcoma: A Case Report and Literature Review”, which was published in *Diagnostics* [1]. The authors described a rare case of synovial sarcoma (SS) that originated in the heart and indicated the importance of pathology and imaging for precise diagnosis. Fluorescence in situ hybridization (FISH) and polymerase chain reaction (PCR) have been essential to the molecular confirmation of the diagnosis of SS; however, in this case, the FISH results were negative. Although there have been a few cases involving negative molecular confirmation results [2], methods of compensating for such diagnostic limitations have been developed [2,3,4]. Therefore, we have introduced other diagnostic tools.

## 2. Commentary

### 2.1. Overview of SS 

SS is a relatively rare type of spindle cell sarcoma with variable epithelial differentiation [5]. SS is considered as a translocation-associated sarcoma because *SS18*::*SSX* fusion has been observed in more than 90% of cases [6]. SS18 genes generally fuse with *SSX1*, *SSX2*, and *SSX4* [6]; however, other fusions including *SS18L1*::*SSX1* and *SS18*::*NEDD4* have been reported as well [2]. More than 70% of SS cases occur in the soft tissues of extremities; however, rare sites including the kidney, bone, lung, and heart have been reported [5,7]. For general SS cases (which are almost the same as cases of SS of the extremities), the peak incidence occurs during the third decade of life, with 90% of cases occurring before the age of 60 years [6]. General SS occurs equally or slightly more often in male patients [5,6]. However, for cardiac SS, the median age is in the third decade of life and it predominantly occurs in male patients [8]. Regarding the prognosis, the 5-year overall survival rate is 79.7% for all types of SS [9]; however, the 1-year and 5-year survival rates for cardiac SS are approximately 59.9% and 29.9%, respectively [10]. Additionally, the patient described by Manole et al. died within 16 months despite surgery and chemotherapy [1]. Complex factors including larger size and R1 and R2 resections are associated with cardiac SS and these may contribute to its poor prognosis [8]. 

### 2.2. Imaging of SS

Characteristics of general SS include the triple sign, pseudo-cystic sign, and calcification [11]. Calcification was observed in 44% of cases using radiography and computed tomography and its presence was associated with a better prognosis [11]; however, it is impossible to differentiate SS from other tumors solely based on calcification because calcification has been observed in various benign and malignant soft tissue tumors. Although the triple sign and pseudo-cystic sign observed using magnetic resonance imaging are characteristics of SS, the rates of positive triple signs and pseudo-cystic signs are relatively low (43% and 22%, respectively) [11]. These three aforementioned features were not detected in the present case. Other magnetic resonance imaging findings, including enhancement, high T2 signal, and the tail sign, were observed in general SS cases [11]; however, unfortunately, these findings are not specific to SS as they are frequently detected in other tumors as well. In terms of positron emission tomography-computed tomography, the maximum standardized uptake value is relatively lower with general SS than with other sarcomas [12]. Thus, the differential diagnosis of SS solely based on imaging findings may be impossible and pathological findings are more conclusive. 

### 2.3. Pathological Findings of SS

#### 2.3.1. Confirmation of SS Using Immunohistochemistry

Although the combination of several antibodies, including CD99, EMA, and S100, has been proposed for diagnosing SS using immunohistochemistry (IHC), it is difficult to obtain the correct diagnosis due to overlap with other benign and malignant tumors [6]. Among the available antibodies, a transducer-like enhancer of split 1 (TLE-1) appears beneficial, with strong diffuse nuclear staining often observed in SS. However, caution is necessary due to the positivity observed in solitary fibrous tumors and malignant peripheral nerve sheath tumors [6]. Additionally, the utility of SMARCB1/INI1 reduction, which exhibits high specificity (100%) and sensitivity (86%), has been reported [13]. To overcome these limitations, SS18-SSX and SSX antibodies have been developed. Zaborowski et al. reported positive rates of 87% and 92%, with specificities of 100% and 93% for SS18-SSX and SSX antibodies, respectively, in SS diagnosis [3]. Additionally, they proposed diagnosing SS based on SSX18-SSX positivity without FISH confirmation [3]. Therefore, IHC using SS18-SSX and SSX antibodies offers great advantages.

#### 2.3.2. Molecular Confirmation of SS

The detection of fusion genes is not absolute but is indispensable for the diagnosis of SS. The sensitivity of FISH for *SS18* is less than 90% [2] and its superiority over PCR remains controversial [2,6]. Decalcification could lead to weak fluorescence and an analysis using FISH was feasible for only 36% of cases of SS of the bone [14]. In addition to FISH and PCR, there are other detection methods, such as RNA-sequencing and fragment analysis [2,4]. The sensitivity of the fragment analysis using capillary electrophoresis was higher than that of FISH and PCR [4]. Careful analyses of rare sites, such as in the present case, as well as the usual sites, are necessary, especially when the FISH results are negative, and several examinations including molecular confirmation by other tools or IHC with SS18-SSX should be performed. Yoshida et al. reported that 11 of 99 cases had negative FISH results (8 cases: negative results; 3 cases: atypical/equivocal results) [2]. Furthermore, among these 11 cases, the diagnosis was enabled by the combination of IHC using SMARCB1, SS18-SSX, and SSX and RNA sequencing [2]. Finally, the differential diagnosis determined by our team was mesothelioma and malignant peripheral nerve sheath tumors. Mesothelioma frequently occurs with cardiac tumors [15]; in particular, sarcomatoid mesothelioma, as described previously, has many similarities to histological and IHC findings of SS. This suggests that differential diagnosis of sarcomatoid mesothelioma and SS may not be possible without molecular confirmation [16]. Characteristics of mesothelioma include loss of BAP1 detected by IHC and deletion of p16 (CDKN2A) detected by FISH [17]. Considering the histological and positive S100 findings, malignant peripheral nerve sheath tumor is an important differential diagnosis in the present case and the deletion of H3K27me3 detected using IHC is a useful marker [18].

## 3. Conclusions

We introduced the development of IHC using SS18-SSX and SSX antibodies and molecular confirmation using tools other than FISH. Although case reports of rare cancer and sarcoma in rare sites are absolutely valuable, an uncertain diagnostic approach will diminish the significance and value of these case reports. Therefore, a variety of diagnostic approaches should be used when possible.

## References

[1] Manole S., Pintican R., Palade E., Duma M.M., Dadarlat-Pop A., Schiau C., Bene I., Rancea R., Miclea D., Manole V. (2022). Primary pericardial synovial sarcoma: A Case report and literature review. Diagnostics.

[2] Yoshida A., Arai Y., Satomi K., Kubo T., Ryo E., Matsushita Y., Hama N., Sudo K., Komiyama M., Yatabe Y. (2022). Identification of novel SSX1 fusions in synovial sarcoma. Mod. Pathol..

[3] Zaborowski M., Vargas A.C., Pulvers J., Clarkson A., de Guzman D., Sioson L., Maclean F., Chou A., Gill A.J. (2020). When used together SS18-SSX fusion-specific and SSX C-terminus immunohistochemistry are highly specific and sensitive for the diagnosis of synovial sarcoma and can replace FISH or molecular testing in most cases. Histopathology.

[4] Shetty O., Pai T., Gurav M., Rekhi B. (2020). Comparison between Fluorescence in-situ Hybridization (FISH), reverse transcriptase PCR (RT-PCR) and fragment analysis, for detection of t (X; 18) (p11; q11) translocation in synovial sarcomas. Indian. J. Pathol. Microbiol..

[5] Suurmeijer A.J.H., Ladanyi M.L.M., Nielsen T.O., WHO Classification of Tumours Editorial Board (2020). Soft Tissue & Bone Tumours.

[6] Thway K., Fisher C. (2014). Synovial sarcoma: Defining features and diagnostic evolution. Ann. Diagn. Pathol..

[7] Ichikawa J., Kawasaki T., Imada H., Kanno S., Ookita G., Taniguchi N., Ashizawa T., Tatsuno R., Jyubashi T., Haro H. (2023). Primary synovial sarcoma of the bone: A case report and literature review. Anticancer. Res..

[8] Coli A., Cassano A., Novello M., Ranelletti F.O., Lauriola L. (2019). Primary cardiac synovial sarcoma: A review correlating outcomes with surgery and adjuvant therapy. J. Card. Surg..

[9] Hagiwara Y., Iwata S., Ogura K., Kawai A., Susa M., Morioka H., Hiruma T., Tsuda Y., Kawano H., Yonemoto T. (2023). Clinical analysis of multimodal treatment for localized synovial sarcoma: A multicenter retrospective study. J. Orthop. Sci..

[10] Wang J.G., Li N.N. (2013). Primary cardiac synovial sarcoma. Ann. Thorac. Surg..

[11] Tordjman M., Honoré C., Crombé A., Bouhamama A., Feydy A., Dercle L., Haddag L., Bouché P.A., Ngo C., Le Cesne A. (2023). Prognostic factors of the synovial sarcoma of the extremities: Imaging does matter. Eur. Radiol..

[12] Sambri A., Bianchi G., Longhi A., Righi A., Donati D.M., Nanni C., Fanti S., Errani C. (2019). The role of 18F-FDG PET/CT in soft tissue sarcoma. Nucl. Med. Commun..

[13] Arnold M.A., Arnold C.A., Li G., Chae U., El-Etriby R., Lee C.C., Tsokos M. (2013). A unique pattern of INI1 immunohistochemistry distinguishes synovial sarcoma from its histologic mimics. Hum. Pathol..

[14] Righi A., Gambarotti M., Benini S., Gibertoni D., Asioli S., Magagnoli G., Gamberi G., Sbaraglia M., Cocchi S., Staals E. (2022). Primary synovial sarcoma of bone: A retrospective analysis of 25 patients. Histopathology.

[15] Maleszewski J.J., Anavekar N.S. (2017). Neoplastic pericardial disease. Cardiol. Clin..

[16] Klebe S., Prabhakaran S., Hocking A., Pulford E., Moore S., Nicola M., Allen P.W., Henderson D.W. (2018). Pleural malignant mesothelioma versus pleuropulmonary synovial sarcoma: A clinicopathological study of 22 cases with molecular analysis and survival data. Pathology.

[17] Hwang H.C., Pyott S., Rodriguez S., Cindric A., Carr A., Michelsen C., Thompson K., Tse C.H., Gown A.M., Churg A. (2016). BAP1 immunohistochemistry and p16 FISH in the diagnosis of sarcomatous and desmoplastic mesotheliomas. Am. J. Surg. Pathol..

[18] Prieto-Granada C.N., Wiesner T., Messina J.L., Jungbluth A.A., Chi P., Antonescu C.R. (2016). Loss of H3K27me3 expression is a highly sensitive marker for sporadic and radiation-induced MPNST. Am. J. Surg. Pathol..

